# Molecularly diverse glycinergic interneurons in the medial deep dorsal horn are interposed between primary sensory afferents and spinal Shox2 interneurons

**DOI:** 10.3389/fncir.2026.1854977

**Published:** 2026-09-03

**Authors:** Jenna R. McGrath, Kimberly J. Dougherty

**Affiliations:** Marion Murray Spinal Cord Research Center, Department of Neurobiology and Anatomy, Drexel University College of Medicine, Philadelphia, PA, United States

**Keywords:** electrophysiology, interneuron, locomotion, sensory afferent, spinal cord

## Abstract

Appropriate transmission of sensory afferent input from the peripheral nervous system to spinal locomotor circuits is crucial for efficient and adaptive locomotion. This is partly mediated by spinal interneurons (INs) that regulate afferent input relayed to locomotor networks. After a spinal cord injury (SCI), sensory afferent feedback becomes critical for motor recovery, however these pathways undergo plasticity. Previously, we observed a loss in the inhibitory sensory afferent pathway to locomotor rhythm generating Shox2 INs after SCI. Importantly, the inhibitory INs within sensory afferent pathways to Shox2 INs have yet to be defined. Thus, the goal of the present study was to identify inhibitory INs interposed between sensory afferents and Shox2 INs in the uninjured mouse. Using electrophysiology and pharmacology to test for connectivity, we demonstrate that lumbar inhibitory INs within sensory afferent pathways to Shox2 INs are glycinergic (GlyT2) INs in the medial deep dorsal horn. Afferent stimulation evoked currents in medial deep dorsal GlyT2 INs that were mostly from excitatory, putative monosynaptic connections. Consistent with the electrophysiology, parvalbumin^+^ afferent fibers densely innervated the region containing the medial deep dorsal GlyT2 INs. Additionally, the medial deep dorsal GlyT2 INs expressed multiple previously described deep dorsal horn molecular markers. These results identify an inhibitory sensory afferent pathway to Shox2 INs comprised of a molecularly diverse population of GlyT2 INs that may be important for the regulation of low-threshold afferent input to the spinal locomotor network.

## Introduction

Sensory information from the periphery is transmitted to the spinal cord via primary afferents and integrated by spinal circuits to adaptively control locomotion (Markin et al., 2010; Rossignol et al., 2006). Activation of proprioceptors and mechanoreceptors in the hindlimb during stepping relay information about physical and environmental stimuli (Frigon et al., 2021; Goulding et al., 2025). Somatosensory feedback contributes to multiple components of locomotion, including phase transitions, interlimb coordination, postural control, and proper paw placement (Frigon et al., 2021; McCrea, 2001). As this input can potentiate, disrupt, or alter locomotion, it is essential that the various inputs are regulated so that relevant information is appropriately processed (Rossignol et al., 2006; Akay et al., 2014; Acevedo and Díaz-Ríos, 2013). In addition to making direct contacts onto motor neurons, sensory afferents synapse onto spinal interneurons (INs) that can act as regulators of sensory information (Jankowska, 2022; Bui et al., 2015). Networks of spinal inhibitory INs play a crucial role in tuning sensory afferent transmission to locomotor circuits through pre- and postsynaptic mechanisms (Bardoni et al., 2013; Stachowski and Dougherty, 2021; Fink et al., 2014; Rudomin and Schmidt, 1999). Precise modulation of sensory afferent input is critical for appropriate sensorimotor behaviors, and targeted silencing of inhibitory INs receiving afferent transmission can manifest as impaired withdrawal reflexes (Hilde et al., 2016; Petitjean et al., 2015) and deficits in locomotor gait (Koch et al., 2017; Gradwell et al., 2024). Therefore, inhibitory INs play a key role in ensuring that accurate sensory information is relayed to locomotor circuits.

While sensory afferent feedback is important for locomotion in uninjured conditions, its role becomes increasingly crucial after a spinal cord injury (SCI) (Takeoka and Arber, 2019; Takeoka et al., 2014; Formento et al., 2018; Bouyer and Rossignol, 2003). Due to the loss of descending drive from the brain, afferent input is the main source of excitation to the spinal cord following SCI (Edgerton et al., 1992; Takeoka, 2020; D’Amico et al., 2014) and blocking this input from entering the spinal cord prevents spontaneous locomotor recovery from occurring (Takeoka and Arber, 2019; Takeoka et al., 2014). On the other hand, activation of primary afferents through rehabilitative strategies such as treadmill training (de Leon and Acosta, 2006; de Leon et al., 1998; Belanger et al., 1996; Cai et al., 2006) and epidural stimulation (Ichiyama et al., 2005; Iwahara et al., 1992; Lavrov et al., 2008; Minassian et al., 2004) results in some locomotor improvements in rodents, cats, and humans. Additionally, a population of INs that integrates sensory afferent feedback has been shown to be critical for locomotor recovery after SCI, even though they are dispensable for normal locomotion (Bui et al., 2016). This demonstrates that sensory afferent feedback, in addition to the INs integrating this feedback, is critical for supporting locomotor recovery after SCI.

Although sensory afferents below the lesion are not directly damaged, plasticity induced by SCI can result in altered sensory afferent transmission. Recently, it has been shown that sensory afferent input to a population of locomotor INs is altered after SCI. Shox2 expressing INs are involved in locomotor rhythm generation in the mouse spinal cord (Dougherty et al., 2013). In response to sensory afferent stimulation, the majority of input to Shox2 INs is minimally disynaptic through interposed excitatory and inhibitory INs in roughly equal proportions (Garcia-Ramirez et al., 2021; Li et al., 2019). After a complete SCI, dorsal root stimulation evokes almost exclusively excitatory potentials in Shox2 INs (Garcia-Ramirez et al., 2021). This suggests a loss of the inhibitory sensory afferent pathway to Shox2 INs after SCI that may be an important restoration point when attempting to promote locomotor recovery.

Despite this, the identity of inhibitory INs within sensory afferent pathways to Shox2 INs remains unknown. Motivated by their possible therapeutic potential to restore locomotor function after SCI, the goal of the present study was to identify the inhibitory INs interposed between primary sensory afferents and Shox2 INs. The functional roles of many spinal neuron populations have been described, with population identity based on the expression of molecular markers (Russ et al., 2021; Stachowski and Dougherty, 2021; Gatto et al., 2019; Goulding et al., 2002). Therefore, we reasoned that determining the expression of known identifying markers in the interposed inhibitory INs would provide insight into the functional relevance of these INs in the uninjured state.

In this study, we demonstrate that Shox2 INs are inhibited by GlyT2GFP INs in the medial deep dorsal horn. The medial deep dorsal horn is a termination zone for sensory afferents (Abraira et al., 2017) and as expected we found that most medial deep dorsal GlyT2GFP INs receive monosynaptic sensory afferent input. Assessing their molecular signature by quantifying the percentage of medial deep dorsal GlyT2GFP INs that overlap with molecular markers of the medial deep dorsal horn, revealed a heterogenous population of GlyT2GFP INs. Taken together, our results describe a molecularly diverse population of GlyT2GFP INs in the medial deep dorsal horn, interposed between sensory afferents and Shox2 INs. Due to their connectivity, this population of inhibitory INs is likely important for appropriate sensory afferent transmission to locomotor circuits.

## Materials and methods

### Mouse lines

Experiments were performed using *Shox2::Cre;R26-lsl-tdTomato;GlyT2GFP*, *Shox2::Cre;R26-lsl-tdTomato;GAD67GFP*, and *RORβ::Cre;R26-lsl-tdTomato;GlyT2GFP* transgenic mice, generated by crossing the following mouse lines: *Shox2::Cre* (Dougherty et al., 2013), *R26-lsl-tdTomato* (Ai9 Jax #007909), (Madisen et al., 2010), *GlyT2GFP* (Zeilhofer et al., 2005), *GAD67GFP* (Jax #003718) (Oliva et al., 2000), and *RORβ::Cre* (Jax #023526) (Harris et al., 2014). All experimental procedures followed National Institutes of Health guidelines and were approved by the Institutional Animal Care and Use Committee at Drexel University.

### Spinal cord preparations

For all spinal cord preparations, mice older than P10 were anesthetized with ketamine (150 mg/kg) and xylazine (15 mg/kg). Mice were decapitated and eviscerated, and spinal cords were removed in ice-cold dissecting solution. The dissecting solution for neonatal mice (P2-10) contained the following (in mM): 111 NaCl, 3 KCl, 11 glucose, 25 NaHCO_3_, 3.7 MgSO_4_, 1.1 KH_2_PO_4_, and 0.25 CaCl_2_. For juvenile mice (P10-P22) the dissecting solution contained (in mM): 222 glycerol, 3 KCl, 11 glucose, 25 NaHCO_3_, 1.3 MgSO_4_, 1.1 KH_2_PO_4_, and 2.5 CaCl_2_. For connectivity experiments, the lumbar spinal cord was sectioned transversely (300–330 μm) in dissecting solution using a vibrating microtome (Leica Microsystems). Slices were immediately transferred to recording artificial cerebrospinal fluid (ACSF) containing the following (in mM): 111 NaCl, 3 KCl, 11 glucose, 25 NaHCO_3_, 1.3 MgSO_4_, 1.1 KH_2_PO_4_, and 2.5 CaCl_2_. Slices from mice older than P14 were incubated at 34 °C–37 °C for 30 min and then at room temperature for 1 h before recording. For dorsal root stimulation experiments, the spinal cord was removed from mice (P4-6) leaving dorsal roots attached. The lumbar spinal cord was hemisected along the midline using a surgical microknife (5.0 mm cutting edge, 15.0 cutting angle, Fine Science Tools #10315-12). Spinal cords were transferred to ACSF and incubated at 34 °C–37 °C for 20 min and then at room temperature for an hour before recording. All dissecting and recording solutions were continuously aerated with 95% O_2_/5% CO_2_.

### Connectivity testing

For connectivity experiments, lumbar spinal slices were prepared from P2-P22 *Shox2::Cre;R26-lsl-tdTomato;GAD67GFP* and *Shox2::Cre;R26-lsl-tdTomato;GlyT2GFP* mice. This age range has been successfully used in prior studies testing connectivity (Ha and Dougherty, 2018). Fluorescently labeled tdTomato^+^ Shox2 INs and GFP^+^ inhibitory neurons were visualized with a 60× objective lens on a BX51WI scope (Olympus) using LED illumination (Lumen Dynamics X-Cite). An electrode connected to a picospritzer was targeted to the somas of closely clustered groups of GFP^+^ GlyT2 or GAD67 INs in the dorsal horn. Picospritzer electrodes were filled with 100 μM kainic acid (Tocris 222) dissolved in water. A Parker Picospritzer III was used to emit kainate at 10–50 PSI for 10–40 ms. The pressure and duration were optimized for each cluster of neurons to show a small physical movement of the neurons upon stimulation. Fluorescently labeled tdTomato^+^ Shox2 INs were targeted for whole-cell patch clamp after optimization of the picospritzer electrode placement and parameters. Electrodes were pulled to tip resistances of 5–10 MΩ using a multi-stage puller (Sutter Instruments) and were filled with intracellular solution. To visualize inhibitory currents, a cesium based intracellular solution was used containing (in mM): 130 CsMeSO_4_, 10 HEPES, 0.5 EGTA, 2 MgCl_2_, 2 ATP, and 5 CsCl. All recordings were performed at room temperature. Shox2 INs were held at −20 mV in voltage clamp and separate recordings were taken in the absence and presence of kainate stimulation of GFP^+^ neurons. Data were collected with a Multiclamp 700B amplifier (Molecular Devices) and Clampex software (pClamp9, Molecular Devices). Signals were digitized at 20 kHz and filtered at 6 kHz. Only one cluster of inhibitory INs was stimulated for each Shox2 IN. When recording from GlyT2 INs directly stimulated with kainate, the latency from the beginning of the stimulation to the peak of the first action potential ranged from 27 to 70 ms (5 neurons). Thus, evoked IPSCs in Shox2 INs with a latency < 90 ms were included as a connected pair. Upon conclusion of the experiment, images of spinal slices with picospritzer and recording electrodes in view were saved (HCImage). The images were overlayed onto a representative neonatal lumbar spinal slice and the tips of the picospritzer and recording electrodes were marked to generate the cell location maps. To quantify the change in frequency and peak amplitude of IPSCs in the presence of kainate, 400 ms of activity was analyzed from 25 sweeps during baseline conditions (absence of kainate) and in the presence of kainate for each GlyT2GFP neuron. The 400 ms kainate window started 40 ms after the beginning of the kainate pulse for recordings in the presence of kainate. Threshold search was performed in Clampfit to detect IPSCs and measure peak amplitude. The mean frequency or amplitude in the presence of kainate was subtracted from the mean frequency or amplitude in the absence of kainate to calculate the delta (Δ).

### Dorsal root stimulation experiments

For dorsal root stimulation experiments, fluorescently labeled GFP^+^ GlyT2 INs in the medial deep dorsal horn of lumbar spinal segments in hemisected isolated spinal cords from *Shox2::Cre;R26-lsl-tdTomato;GlyT2GFP* mice at P4-P6 were targeted for whole-cell patch clamp recordings as described above. This age range was used because the viability of the hemisected spinal cord preparation decreases with age. The intracellular solution contained (in mM): 128 K-gluconate, 10 HEPES, 0.0001 CaCl_2_, 1 glucose, 4 NaCl, 5 ATP, and 0.3 GTP. Glass suction electrodes used to stimulate dorsal roots (L2-L5) were pulled to tip resistances of 5–10 MΩ using a multi-stage puller (Sutter Instruments) and sanded to a wider tip circumference to fit the dorsal rootlet. Electrode holders (Warner Instruments 64–1045) were connected to a stimulator (Universal Stimulus Isolator Model 401, University of Cologne) and electrical stimulation was delivered by a 0.1–0.2 ms pulse at synaptic response threshold (20–170 μA) and then at 1.5, 2 and 5× threshold intensity (max 500 μA) with a minimum of 30 s between trials. Voltage clamp recordings at −60 mV and −30 mV, each for at least 15 trials, were done to detect the presence of a dorsal root stimulation evoked post-synaptic current. Some cells are represented more than once in the data because their response was recorded when stimulating multiple roots. Latency was measured from the beginning of the dorsal root stimulation to the beginning of the evoked current.

### Immunohistochemistry

In some electrophysiology experiments, biocytin (2 mg/mL, Sigma-Aldrich B4261) was included in the intracellular solution. After completion of recordings, slices containing biocytin-filled neurons were placed into a 4% PFA solution overnight. They were subsequently placed in PBS at 4 °C. To visualize biocytin, the slices were incubated for 2 h at room temperature in DyLight 633 conjugated streptavidin (1:400, Thermo Fischer Scientific 21844). Images were acquired at 10× on a Leica DM6 LED epifluorescence microscope and processed with THUNDER imaging software.

Spinal cords from *RORβ::Cre;lsl-tdTomato;GlyT2GFP* and *Shox2::Cre;lsl-tdTomato;GlyT2GFP* mice at P4 and P7 were harvested and immediately placed into a 4% PFA solution and fixed overnight at 4 °C. These ages were chosen because they correspond with the electrophysiology connectivity and because some of the transcription factors tested are downregulated with age. *RORβ::Cre;lsl-tdTomato;GlyT2GFP* mice at P20 were anesthetized with ketamine (150 mg/kg) and xylazine (15 mg/kg) and perfused transcardially with 0.1M PBS, followed by 4% PFA in PBS. This tissue was used to quantify parvalbumin neurons, as interneurons begin to express parvalbumin around P10. Cords were harvested and post-fixed at 4 °C overnight and subsequently transferred to 30% sucrose in PBS for at least 48 h. Tissue was embedded in OCT compound (Thermo Fisher Scientific) over dry ice and stored at −80 °C. Spinal cords were sectioned at 20 μm transversely on a cryostat (Microm HM 550) and directly mounted onto slides.

Slides were washed with PBS and then blocked in a PBS solution containing either 5% donkey or goat serum, 1% bovine serum albumin, 0.2% Triton X-100, and 0.1% fish gelatin. When using the mouse anti-Prox1 antibody, slides were then blocked in a mouse IgG blocking reagent (Vector Laboratories, MKB-2213-1) for 1 h. Slides were incubated overnight with one of the following antibodies: rabbit anti-Satb2 (1:800, Abcam AB34735), rabbit anti-Tfap2b (1:750, Novus NBP1-89063), rabbit anti-Parvalbumin (1:750, Swant PV27a), or mouse anti-Prox1 (1:400, Millipore MAB5654). Slides were then incubated for 2 h in one of the following secondary antibodies at 1:400: goat anti-rabbit 647 (Invitrogen A-21245), donkey anti-rabbit 647 (Jackson 711-605-152) or donkey anti-mouse 647 (Invitrogen A-31571). All immunohistochemistry steps were performed at room temperature. All slides were coverslipped using Fluoromount-G (Vector Laboratories, H-1000-10). Images were acquired as sequential z-stacks of 40× tile-scans on a Leica DM6 LED epifluorescence microscope. Cell counting was performed manually in ImageJ using the multipoint tool. The deep dorsal horn was defined by a rectangular region of interest that was scaled to each spinal slice. The boundaries of the rectangles are as follows: dorsal – bottom of lamina III, ventral – top of the central canal, medial – edge of dorsal column, lateral – half the distance from central canal to edge of slice.

### Statistical analyses

Statistical tests and *post hoc* analyses were performed with GraphPad Prism (GraphPad Software). All results are presented as mean ± SD. Statistical significance was set at *p* < 0.05. The distribution of the data was determined by Shapiro-Wilk normality test. The Wilcoxon matched-pairs test was used to compare before and after kainate conditions. The Mann-Whitney test was used to compare the changes in frequency and amplitude of IPSCs in Shox2 INs between GlyT2GFP-Shox2 INs connected and non-connected pairs. The Kruskal-Wallis with Dunn’s multiple comparisons *post-hoc* test was used to compare the latencies of the 3 response types evoked by dorsal root stimulation.

## Results

### GFP^+^ INs have minimal spatial overlap in the dorsal horn of GAD67GFP and GlyT2GFP mice

**FIGURE 1:**
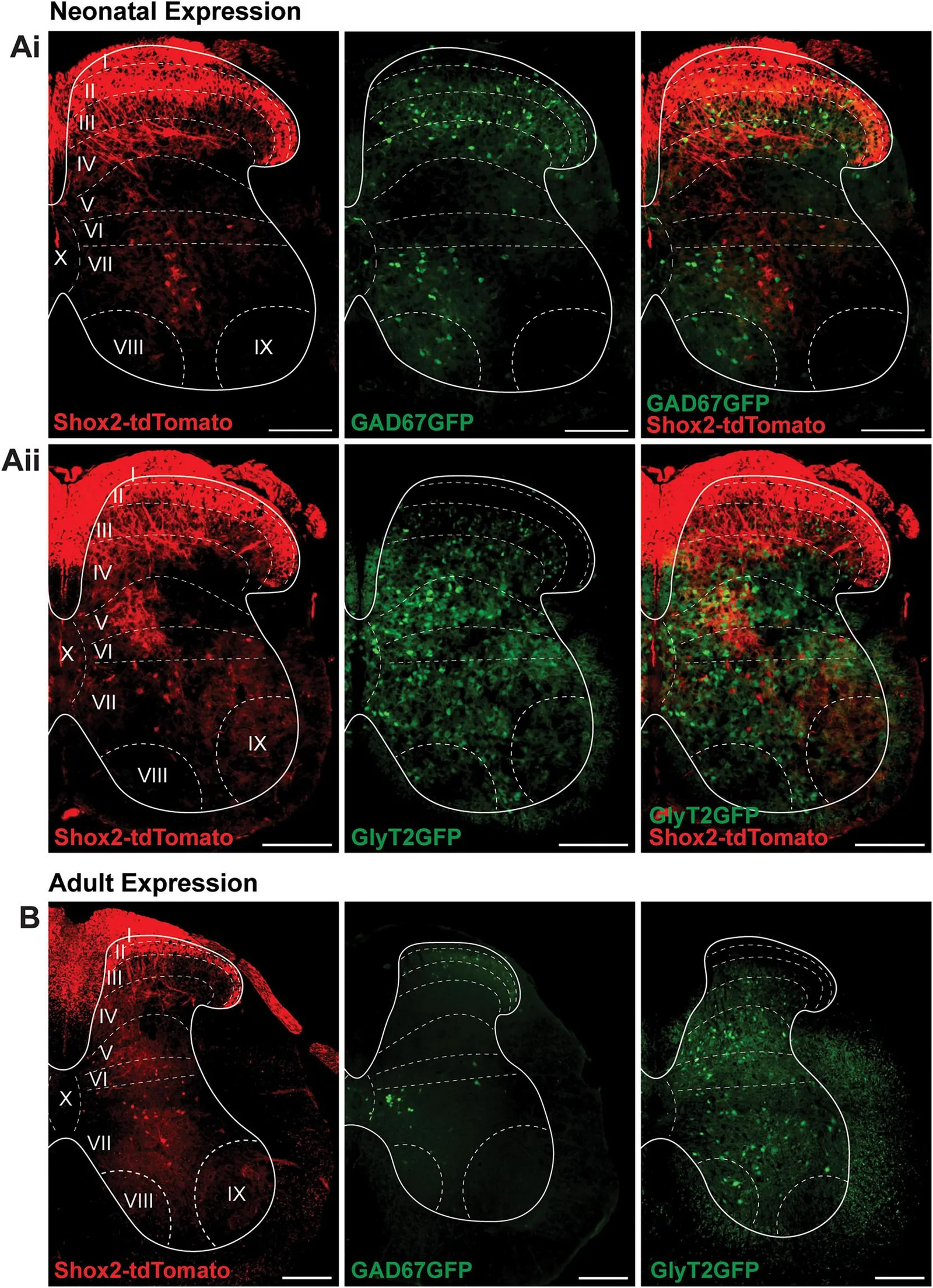
GFP^+^ neurons have minimal spatial overlap at neonatal and adult ages in GAD67GFP and GlyT2GFP mouse lines. **(Ai,Aii)** Representative images of the expression patterns of fluorescently labeled neurons within the spinal cord laminae (I–X) for *Shox2::Cre;R26-lsl-tdTomato;GAD67GFP* **(Ai)** and *Shox2::Cre;R26-lsl-tdTomato*;*GlyT2GFP* **(Aii)** mouse lines. **(B)** The expression patterns of fluorescently labeled neurons remain consistent throughout adulthood for all 3 neuron types. Note the minimal spatial overlap of GFP^+^ neurons in the dorsal horn. Dorsal horn tdTomato labeling is due to Shox2 expression in primary afferents. Scale bars are 200 μm.

Subsets of Shox2 INs have been shown to receive inhibitory input in response to dorsal root stimulation in both the neonatal and adult mouse (Garcia-Ramirez et al., 2021; Li et al., 2019). In order to determine the identity of the inhibitory INs that provide input to Shox2 INs, Shox2::Cre;lsl-tdTomato;GlyT2GFP and Shox2::Cre;lsl-tdTomato;GAD67GFP mouse lines were used to visualize either glycinergic (GlyT2) or GABAergic (GAD67) neurons respectively, and Shox2 INs in the same lumbar spinal slice. We performed our connectivity testing in younger mice to increase feasibility and yield. We first assessed the distributions of Shox2 and inhibitory INs in neonatal and adult mice. At both ages, most Shox2 INs are located in a band that extends mediolaterally at the level of the central canal in lamina VII (Figures 1A, B; Dougherty et al., 2013; Garcia-Ramirez et al., 2021). In neonatal GAD67GFP mice, GFP^+^ INs populate throughout the mediolateral extent of laminae I-IV with very few within lamina V-VI. There is also a high density of GAD67GFP INs medially in lamina VII, VIII and X (Figure 1Ai; Dougherty et al., 2009). GlyT2GFP INs are sparsely present in lamina I and have a higher density throughout laminae III-VIII and X (Figure 1Aii; Restrepo et al., 2009). There is a prominent population of GlyT2GFP INs in the deep dorsal horn (V/VI) where GAD67GFP INs are largely absent (Figure 1A). The expression patterns of GAD67GFP and GlyT2GFP INs appear to remain stable in the adult mouse (Figure 1B) and have been previously reported (Heinke et al., 2004; Oliva et al., 2000; Zeilhofer et al., 2005; Dougherty et al., 2009). Notably, there is little spatial overlap of GFP^+^ INs in GAD67GFP and GlyT2GFP mouse lines in the lumbar dorsal horn.

### Shox2 INs receive inhibitory input primarily from GlyT2GFP INs in the medial deep dorsal horn

**FIGURE 2:**
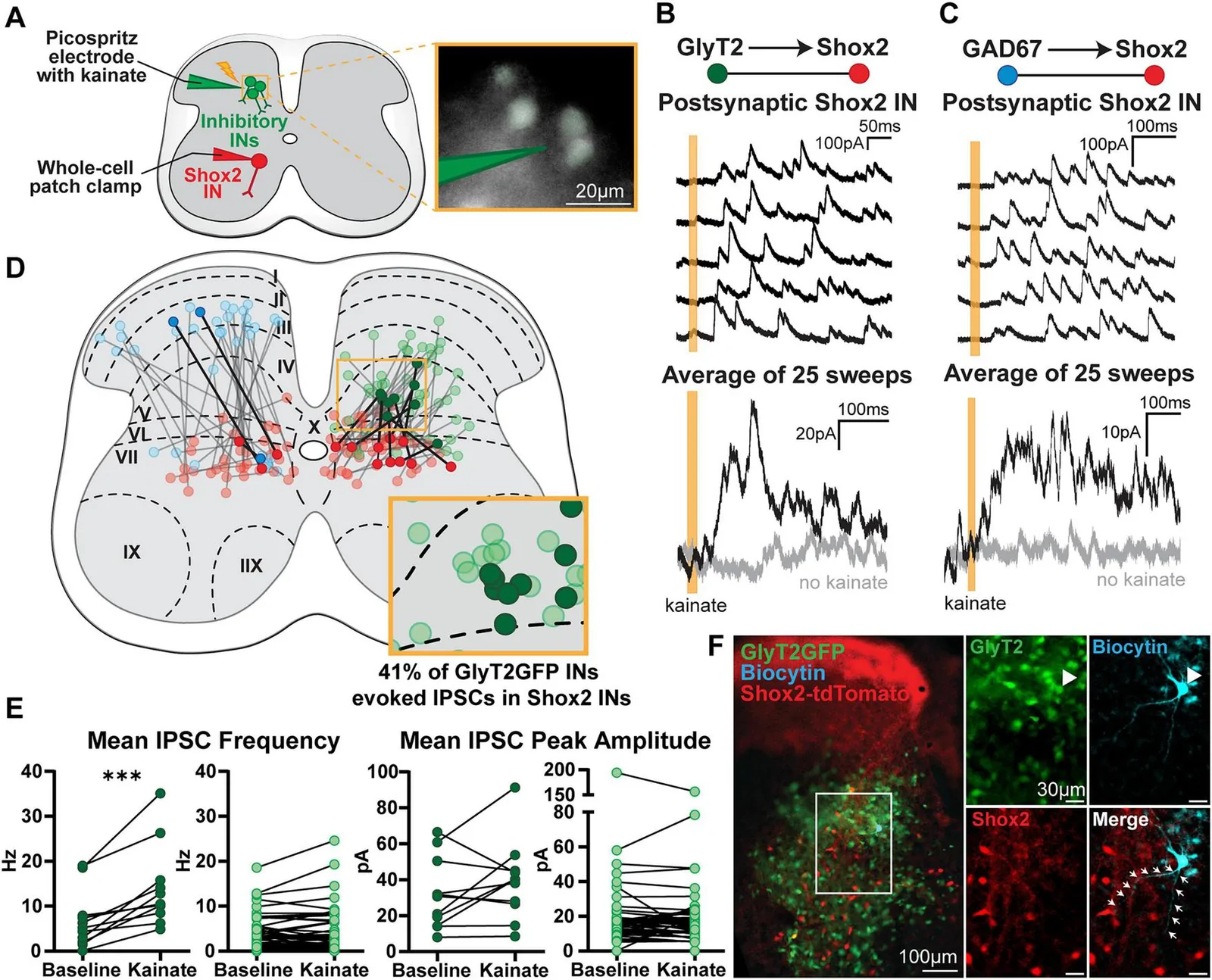
Shox2 INs primarily receive inhibitory input from GlyT2GFP INs in the medial deep dorsal horn. **(A)** Experimental setup displaying the picospritzer electrode used to stimulate a cluster of GFP^+^ inhibitory INs and a recording electrode used for whole-cell patch clamp recordings from a Shox2 IN. The inset image displays the placement of the picospritzer electrode and a cluster of GFP^+^ neurons at 60X. **(B,C)** Examples of Shox2 IN recordings during connectivity testing. Top: Individual sweeps of voltage clamp recordings at –20 mV from a Shox2 IN when a cluster of GlyT2 INs **(B)** or GAD67 INs **(C)** is stimulated using kainate. The duration of the kainate stimulation is denoted by the orange bar. Bottom: The average of trials recording spontaneous activity of the Shox2 IN in the absence of kainate (gray trace) is shown in slices from mice with labeled GFP^+^ GlyT2 INs **(B)** or GAD67 INs **(C)**. The mean of evoked IPSCs in a Shox2 IN from kainate stimulation of GFP^+^ GlyT2 INs **(B)** or GAD67 INs **(C)** is shown in the black trace. **(D)** A representative neonatal spinal slice showing the location of the stimulated GAD67GFP INs (blue, left side) and GlyT2GFP INs (green, right side) and the paired Shox2 IN (red) recorded. Darker symbols indicate pairs of inhibitory INs and Shox2 INs where an evoked IPSC was recorded in the Shox2 IN. Lighter symbols indicate tests without an evoked IPSC. Inset shows stimulated GlyT2 INs in the medial deep dorsal horn. 9/22 (41%) GlyT2GFP IN tests evoked an IPSC in a Shox2 IN. **(E)** Comparisons of the mean IPSC frequency and peak amplitude in Shox2 INs in the absence (baseline) and presence of kainate for connected GlyT2GFP-Shox2 IN pairs (left, dark green symbols), and non-connected pairs (right, light green symbols). The frequency of IPSCs is significantly higher in the presence of kainate for connected pairs (***p = 0.001) compared to non-connected pairs (*p* = 0.626). The mean peak amplitude of spontaneous IPSCs (baseline) and IPSCs in the presence of kainate observed in Shox2 INs does not significantly differ for connected pairs (*p* = 0.278) and non-connected pairs (*p* = 0.102). **(F)** A medial deep dorsal GlyT2GFP IN filled with biocytin, reveals its processes projecting ventrally toward Shox2 INs. Inset image denotes the biocytin-filled GlyT2GFP IN with an arrowhead, and the arrows point to its processes that project to lamina VII, near many Shox2 INs.

To test for connections between either GAD67GFP or GlyT2GFP INs and Shox2 INs in lumbar spinal slices, small clusters of inhibitory GFP^+^ INs were activated pharmacologically with kainate, while simultaneously recording from a Shox2 IN (Figure 2A). In some tests, activation of the GFP^+^ INs evoked IPSCs in the Shox2 IN (Figures 2B, C). GAD67GFP INs were activated in laminae I-II (*n* = 4), laminae III-VI (*n* = 21), laminae V-VI (*n* = 1) and lamina VII (*n* = 10). Only 8% (3 of 36) of GAD67GFP IN tests elicited kainate-evoked inhibitory currents in a Shox2 IN. GlyT2GFP INs were activated in laminae III-IV (*n* = 14), laminae V-VI (*n* = 32) and lamina VII (*n* = 10), but not in the superficial dorsal horn due to their absence from that region. 20% (11 of 56) of GlyT2GFP IN tests elicited a kainate-evoked inhibitory current in a Shox2 IN.

The location of the activated GAD67GFP and GlyT2GFP INs, along with the Shox2 INs associated for each test, were mapped onto a representative lumbar spinal slice to visualize regions of the spinal cord containing connected inhibitory neurons (Figure 2D). GAD67GFP INs with input to Shox2 INs were located in lamina III (*n* = 2) or lamina VII (*n* = 1). GlyT2GFP INs that inhibit Shox2 INs were concentrated in the medial portion of lamina V. In that region, there was a higher percentage of GlyT2GFP INs (41%, 9 of 22 tests) with connections to Shox2 INs than in any other individual region, or the other regions combined (Figure 2D). The average latency of evoked IPSCs from the medial deep dorsal horn was 54 ms ± 15. This falls within the range of action potential latencies (27–70 ms) seen when recording from an inhibitory IN directly stimulated with kainate. Due to the relatively short latency of the initial responses and the fact that an IPSC observed must be due to an inhibitory neuron with direct input, it is likely that these connections are monosynaptic.

Almost all of the Shox2 INs that we recorded from displayed spontaneous IPSCs during baseline conditions, before the application of kainate. Therefore, to validate the absence/presence of connections, we compared the mean frequency of spontaneous IPSCs in Shox2 INs before application of kainate and with the kainate activation. Tested pairs where GlyT2GFP INs evoked an IPSC in a Shox2 IN, had a significantly higher mean IPSC frequency in the presence of kainate (baseline: 6.9 ± 6.4 Hz, kainate: 14.1 ± 9.0 Hz, Wilcoxon matched-pairs test, *p* = 0.001), compared to those that did not evoke an IPSC in a Shox2 IN (baseline: 4.0 ± 4.1 Hz, kainate: 4.3 ± 5.2 Hz, Wilcoxon matched-pairs test, *p* = 0.626) (Figure 2E). Additionally, the mean change in frequency of IPSCs was significantly higher in Shox2 INs where stimulation of GlyT2GFP INs evoked an IPSC (7.2 ± 4.8 Hz), compared to those that did not (0.3 ± 1.9 Hz, Mann-Whitney test, *p* < 0.0001). For both pairs of GlyT2GFP INs that evoked an IPSC in a Shox2 IN (baseline: 31.8 ± 19.6 pA, kainate: 39.1 ± 22.0 pA, Wilcoxon matched-pairs test, *p* = 0.278) and those that did not (baseline: 21.9 ± 28.9 pA, kainate: 20.9 ± 24.7 pA, Wilcoxon matched-pairs test, *p* = 0.052), there is no significant difference in the mean peak amplitude per sweep (Figure 2E). There was also no difference in the mean change in peak amplitude of IPSCs in Shox2 INs for connected (7.3 ± 17.6 pA) and non-connected pairs (−1.0 ± 9.4 pA; Mann-Whitney test, *p* = 0.227).

In other experiments, a medial deep dorsal GlyT2GFP IN was filled with biocytin to visualize where its processes project in the spinal cord. The processes from a biocytin filled GlyT2GFP IN can be seen projecting ventrally toward lamina VII where many Shox2 INs reside (Figure 2F). Given the connectivity rate between medial deep dorsal GlyT2GFP INs and Shox2 INs, and the anatomical evidence of projections from GlyT2GFP INs to Shox2 INs, we conclude that lumbar Shox2 INs receive inhibitory input from GlyT2GFP INs in the medial deep dorsal horn.

### Most GlyT2GFP INs in the medial deep dorsal horn receive monosynaptic input from primary sensory afferents

**FIGURE 3:**
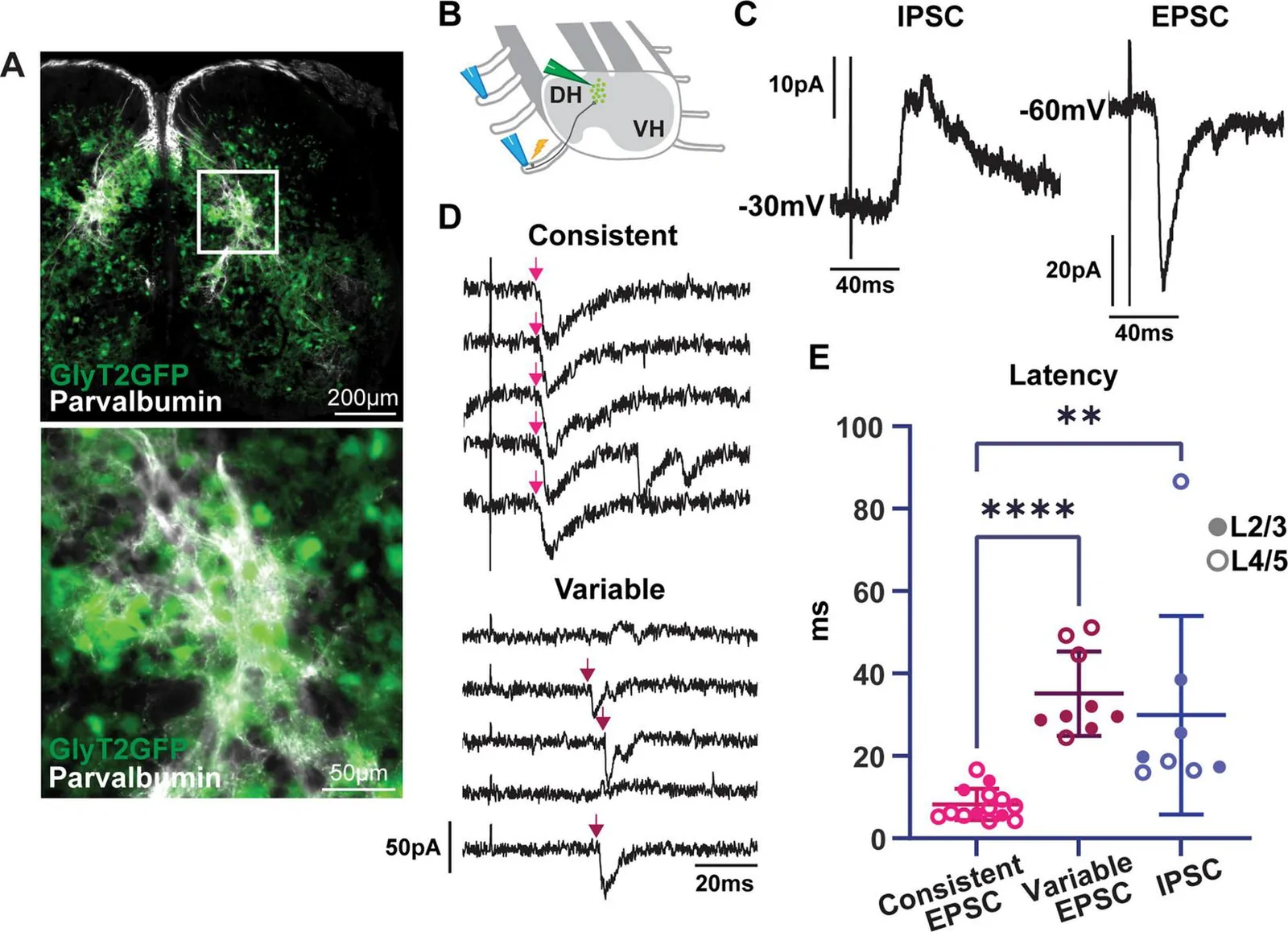
Medial deep dorsal GlyT2GFP INs receive direct sensory afferent input. **(A)** Parvalbumin antibody staining of low-threshold afferents shows robust overlap with medial deep dorsal GlyT2GFP INs. **(B)** Schematic of hemisected neonatal spinal cord preparation where GlyT2GFP INs were recorded from in the dorsal horn (DH) while lumbar dorsal roots were electrically stimulated. **(C)** Left: Example of a mean evoked IPSC from a medial deep dorsal GlyT2GFP IN during voltage clamp recording at –30 mV. Right: Example of a mean dorsal root stimulation evoked EPSC from a medial deep dorsal GlyT2GFP IN during voltage clamp recording at –60 mV. **(D)** Individual sweeps from a medial deep dorsal GlyT2GFP IN where dorsal root stimulation evoked an EPSC that was consistent (top) or variable (bottom) throughout sweeps. The arrows denote the beginning of the EPSC within each sweep. **(E)** The latency of the currents for consistent EPSC, variable EPSC, and IPSC response types. Open circles are responses to L4/L5 dorsal root stimulation and closed circles are responses to L2/L3 dorsal root stimulation. For some cells, both L2/3 and L4/5 roots were stimulated in turn. Consistent EPSC response types have a significantly shorter latency than variable EPSC (****p < 0.0001) and IPSC response types (**p = 0.0053). Kruskal-Wallis with Dunn’s *post-hoc* test.

Neurons within the deep dorsal horn of the spinal cord receive multimodal sensory afferent input from proprioceptors and low threshold mechanoreceptors (Jankowska, 1992; Koch et al., 2018; Gradwell et al., 2024). Parvalbumin is a marker for proprioceptive afferents (Siembab et al., 2010) and a small population of low threshold mechanoreceptors (de Nooij et al., 2013). Parvalbumin antibody staining was performed to visualize projection patterns in relation to GlyT2GFP INs in the medial deep dorsal horn. Consistent with prior reports (Alvarez et al., 2005), we found robust parvalbumin labeling in same region as medial deep dorsal GlyT2GFP INs, suggesting that the these GlyT2GFP INs receive low threshold sensory afferent input (Figure 3A).

We directly tested for afferent input to medial deep dorsal GlyT2GFP INs using *ex vivo* electrophysiology. Medial deep dorsal GlyT2GFP INs in lumbar segments L2 to L5 were recorded from isolated hemisected neonatal spinal cords while stimulating lumbar dorsal roots (Figure 3B). All GlyT2GFP INs (21 of 21 neurons) recorded in the medial deep dorsal horn responded to dorsal root stimulation. To determine if medial deep dorsal GlyT2GFP INs receive input from multiple segments, in some preparations, we stimulated a rostral lumbar (L2 or L3) and a caudal lumbar (L4 and L5) dorsal root, in turn, while recording from a single GlyT2GFP IN. 83% (10 of 12 neurons) responded to dorsal root stimulation of both roots. The remaining two INs responded to dorsal root stimulation of either the rostral or caudal lumbar root (17%, 2 of 12 neurons). In cases where a neuron responded to dorsal root stimulation of a lumbar root, we examined the earliest component of the evoked response. Most of the earliest responses to dorsal root stimulation seen in medial deep dorsal GlyT2GFP INs (74%, 23 of 31) were EPSCs (Figure 3C). In other cases (26%, 8 of 31), the earliest component was an IPSC (Figure 3C). The mean latency for the earliest component was measured, and for EPSCs, the latency and consistency of the EPSC was assessed throughout individual sweeps to determine the likelihood of monosynaptic connection. We found that 61% (14 of 23) of evoked EPSCs in medial deep dorsal GlyT2GFP INs had a consistent latency and no failures. The remaining evoked EPSCs (39%, 9 of 23) showed a variable response latency and/or a failure to respond in some sweeps (Figure 3D). Consistent EPSCs had a significantly shorter latency (8.2 ± 3.8 ms) than both EPSCs with inconsistent latencies (35.1 ± 10.3 ms; Kruskal-Wallis test: *p* < 0.0001) and IPSCs (29.9 ± 24.10 ms; Kruskal-Wallis test: *p* = 0.0053) (Figure 3E). This suggests that the majority of EPSCs evoked from dorsal root stimulation are monosynaptic. Importantly, we found that 57% (12 of 21 neurons) of medial deep dorsal GlyT2GFP INs received monosynaptic input from at least one dorsal root. These data show that while medial deep dorsal GlyT2GFP INs receive sensory afferent input through excitatory and inhibitory pathways, most receive monosynaptic input from primary afferents.

### GlyT2GFP INs in the medial deep dorsal horn overlap with multiple molecular markers

The dorsal horn of the spinal cord is comprised of distinct populations of INs that can be identified by specific molecular markers (Stachowski and Dougherty, 2021; Koch et al., 2018; Gatto et al., 2019). Known molecular markers that label inhibitory INs in the medial deep dorsal horn include RORβ (Koch et al., 2017), Prox1 (Sweeney et al., 2018), Satb2 (Hilde et al., 2016), parvalbumin (Gradwell et al., 2024), and Tfap2b (Levine et al., 2014). None of these markers are exclusive to the medial deep dorsal horn, but they have been shown to be expressed in this region. To determine the extent to which GlyT2GFP INs overlap with each of the five markers previously described, the percentage of neurons within the medial deep dorsal horn expressing one or both markers was quantified.

**FIGURE 4:**
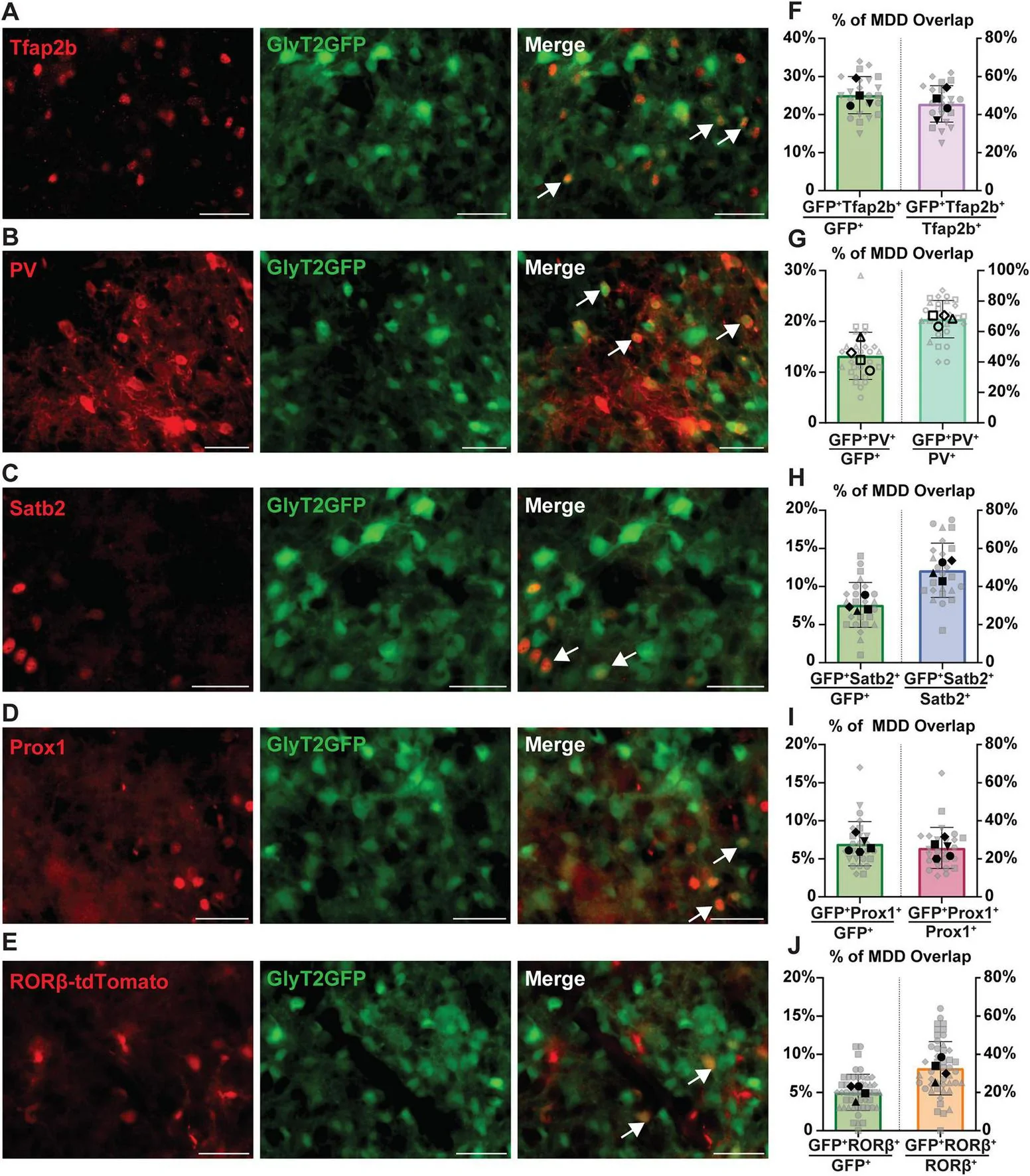
Medial deep dorsal (MDD) GlyT2GFP INs overlap with multiple molecular markers of the deep dorsal horn. **(A–E)** Representative images of the medial deep dorsal horn demonstrating overlap of GlyT2GFP INs with Tfap2b **(A)**, parvalbumin (PV) **(B)**, Satb2 **(C)**, Prox1 **(D)**, and RORβ **(E)**. Neurons expressing both GFP and the molecular marker are indicated with arrows. Scale bars are 50 μm. **(F–J)** The extent of the co-expression of GFP and Tfap2b **(F)**, PV **(G)**, Satb2 **(H)**, Prox1 **(I)**, or RORβ **(J)** in the medial deep dorsal horn. Bar graphs show the percentage of medial deep dorsal GlyT2GFP INs that express a molecular marker on the left, and the percentage of medial deep dorsal neurons positive for each molecular marker that expresses GFP on the right. Note the difference in the *y*-axis scaling on left and right sides. Black symbols indicate mean for each animal and gray symbols indicate individual spinal slices quantified. Each animal is indicated by a distinct symbol, with open symbols representing animals at P20 **(G)**. Supplementary Figure 1 contains images of the full sections that the deep dorsal horn was cropped from for this figure.

Tfap2b^+^ neurons were found throughout the medial-lateral extent of the deep dorsal horn with sparse expression in the superficial dorsal horn and the ventral horn, matching previous characterization (Supplementary Figure 1A; Levine et al., 2014). Tfap2b had the most overlap with medial deep dorsal GlyT2GFP INs compared to the other four markers with 25% ± 3% expressing both Tfap2b and GFP (Figures 4A, F) (*N* = 4, *n* = 22 slices). Of the Tfap2b INs in the medial deep dorsal horn, 48% ± 10% expressed GFP (Figure 4F).

Parvalbumin expression within INs first appears at P10 and increases with development (Siembab et al., 2010), therefore, staining was performed in mice at P20. Consistent with previous reports, we found parvalbumin^+^ neurons in the superficial (Hughes et al., 2012) and deep dorsal horn (Gradwell et al., 2024), as well as in the ventral horn (Supplementary Figure 1B; Alvarez et al., 2005; Siembab et al., 2010). 13% ± 3% of the GlyT2GFP INs in the medial deep dorsal horn expressed parvalbumin and of the parvalbumin^+^ INs in the medial deep dorsal horn, 68% ± 3% expressed GFP (Figures 4B, G) (*N* = 4, *n* = 26 slices).

Aligning with previous work (Hilde et al., 2016), we found that Satb2 is expressed throughout the mediolateral axis of the deep dorsal horn (Supplementary Figure 1C). 8% ± 1% of GlyT2GFP INs in the medial deep dorsal horn expressed Satb2 and of the Satb2 INs in the medial deep dorsal horn, 49% ± 5% expressed GFP (Figures 4C, H) (*N* = 4, *n* = 24 slices).

Prox1^+^ neurons were found throughout the superficial dorsal horn and were sparsely present in the ventral horn. In the deep dorsal horn, Prox1^+^ neurons were predominantly located medially near the central canal (Supplementary Figure 1D). 7% ± 1% of GlyT2GFP INs in the medial deep dorsal horn expressed Prox1 and of the Prox1^+^ INs in the medial deep dorsal horn, 25% ± 5% also expressed GFP (Figures 4D, I) (*N* = 5, *n* = 25 slices).

RORβ neurons are present throughout the superficial dorsal horn with a prominent population in lamina III and IV. There is a smaller population of RORβ neurons in the deep dorsal horn that are medially concentrated (Supplementary Figure 1E). This expression pattern is consistent with prior reports (Del Barrio et al., 2013; Koch et al., 2017; Stachowski et al., 2025). RORβ neurons constituted the smallest proportion of medial deep dorsal horn GlyT2GFP INs, with 5% ± 1% of medial deep dorsal horn GlyT2GFP INs expressing tdTomato, indicating RORβ expression (Figures 4E, J) (*N* = 4, *n* = 46 slices). Of the RORβ neurons in the medial deep dorsal horn, 32% ± 6% also expressed GFP (Figure 4J). Antibodies against the other tested markers (Tfap2b, Satb2, Prox1 and parvalbumin) were applied to spinal tissue from the RORβ::Cre;lsl-tdTomato;GlyT2GFP mice to assess co-expression in the medial deep dorsal horn. RORβ neurons did not express Satb2, and very few expressed parvalbumin (6% of RORβ neurons were parvalbumin^+^ and 4% of parvalbumin^+^ neurons were tdTomato^+^) and Prox1 (3% of RORβ neurons were Prox1^+^ and 1% of Prox1^+^ neurons were tdTomato^+^), consistent with previous findings (Gradwell et al., 2024; Koch et al., 2017). We did, however, find that about half (53%) of the medial deep dorsal RORβ neurons express Tfap2b and 16% of the medial deep dorsal Tfap2b^+^ neurons were tdTomato^+^ neurons (*N* = 3, *n* = 15 slices). Further, 51% ± 7% of neurons expressing both tdTomato and Tfap2b were GlyT2GFP INs (Supplementary Figure 2). This demonstrates that there is a sub-population of medial deep dorsal GlyT2GFP INs that contain both Tfap2b and RORβ molecular markers.

Taken together, medial deep dorsal GlyT2GFP INs are a heterogeneous population, comprised of Tfap2b, Satb2, parvalbumin, Prox1, and RORβ expressing neurons. Notably, all of these markers are expressed outside of the medial deep dorsal horn, so those overlapping with medial deep dorsal GlyT2GFP INs are only a small proportion of the total population. Each marker is expressed at varying degrees within subpopulations of medial deep dorsal GlyT2GFP INs, with Tfap2b being most commonly co-expressed.

## Discussion

Our results demonstrate that lumbar Shox2 INs receive inhibitory input from GlyT2GFP INs located in the medial deep dorsal horn. Further, we show that medial deep dorsal GlyT2GFP INs receive direct sensory afferent input and are within an area densely innervated by proprioceptive afferent terminals. This population of GlyT2GFP INs is heterogenous, with subsets of neurons expressing five previously described molecular markers for dorsal horn IN populations, including Tfap2b, parvalbumin, Satb2, Prox1, and RORβ. Thus, we have identified a population of GlyT2GFP INs defined by circuit connectivity, rather than the expression of a specific molecular marker.

### Identification of the source of inhibitory input to Shox2 INs

Transgenic mouse lines allow for the identification of specific neuron populations and the GFP^+^ neurons in the GlyT2GFP and GAD67GFP mouse lines align with the known distributions of glycinergic (Hossaini et al., 2007; van den Pol and Gorcs, 1988) and GABAergic (Heinke et al., 2004) neurons in the spinal cord, respectively. Despite this, GFP^+^ neurons in these mouse lines constitute only a portion of the total inhibitory populations. It has been noted that glycinergic neurons in lamina II lack somata GFP expression in the GlyT2GFP mouse (Zeilhofer et al., 2005), and about two thirds of the total GABAergic population expresses GFP in the GAD67GFP mouse (Dougherty et al., 2009). GFP neurons in the GAD65GFP mouse line have also been described to be in the deep dorsal horn (Wilson et al., 2010; Betley et al., 2009), but they were not tested in our study. While they were not included in our experiments, there is high overlap between GAD67 and GAD65 (Mackie et al., 2003), and many GFP^+^ terminals in the GAD65GFP mouse line are GAD67^+^ (Wilson et al., 2010). It is likely that some of the GFP expressing neurons in the GAD67GFP mouse line include those labeled in the GAD65GFP line. GFP^+^ terminals in the GAD65GFP mouse line rarely co-expressed GlyT2 (Wilson et al., 2010) and the positions of the GFP^+^ neurons in the dorsal horns of GAD67GFP and GlyT2GFP mouse lines are relatively discrete. However, some inhibitory INs co-release GABA and glycine (Jonas et al., 1998; Mackie et al., 2003) so there is likely to be more co-expression between the transmitters than there appears to be when comparing the mouse lines. Therefore, the lines used here likely encompass a large proportion of the inhibitory neurons in the spinal cord but there may be some dorsal inhibitory subpopulations that were not directly tested.

The majority of the connections between spinal IN populations is hypothesized based on models that can reproduce experimental data (Rybak et al., 2015; Danner et al., 2019) with well-documented exceptions (Alvarez et al., 2013; Hultborn et al., 1971). IN populations are intermingled, rather than in strict lamina or nuclei, which complicates the use of tracers to determine connectivity of local spinal INs. However, Rabies-based tracing has been successful in identifying presynaptic connections to ventral INs (Vieillard et al., 2023; Chapman et al., 2025). A recent study using this strategy revealed neurons presynaptic to Shox2 INs in the adult mouse, including a high density region in the deep dorsal horn (Singh et al., 2026). The identity of these presynaptic neurons was not investigated but supports our finding of presynaptic GlyT2GFP INs in this region.

Testing for connectivity using direct electrophysiological testing is difficult and low yield (Ha and Dougherty, 2018). Thus, a strategy that has proved effective and more efficient is local activation of glutamate receptors (Chopek et al., 2018; Dougherty et al., 2013; Jonas et al., 1998). Since the inhibitory INs were clustered, we used this to our advantage by puffing kainate at a pressure that activated glutamate receptors on a small number of inhibitory INs in close proximity, rather than singular INs, during each test. While it is a more efficient method, the stimulation is less specific, resulting in activation of any neuron (soma or dendrite) exposed to a sufficient kainate concentration. The stimulation is not specific to neuron type, which can complicate testing excitatory connections since it is difficult to show that an EPSC is resulting from the neuron activated, rather than an indirect activation. This is less of an issue with inhibitory connections since any IPSCs evoked in Shox2 INs must be the result of the activation of the soma or dendrite of an inhibitory IN with direct input. There are also excitatory INs interposed between sensory afferents and Shox2 INs (Garcia-Ramirez et al., 2021; Li et al., 2019), however, their location in the spinal cord is unknown. We observed some kainate-evoked EPSCs during connectivity testing of both GlyT2GFP and GAD67GFP INs and Shox2 INs, but EPSCs were never evoked when stimulating neurons in medial lamina V (Supplementary Figure 3), suggesting that the excitatory neurons in this pathway are in a different region. We cannot, however, rule out that we are activating polysynaptic pathways that result in the inhibitory currents seen in Shox2 INs, but this can only occur if the activation of an excitatory neuron evokes an action potential in an inhibitory neuron, which then evokes an IPSC in a Shox2 IN. In our pairwise testing using this strategy [both here and in Dougherty et al. (2013)], we never evoked an action potential in a postsynaptic neuron. Nonetheless, to mitigate this possibility, we included neurons only with a short latency inhibitory response to the kainate stimulation, relative to the time of the action potential peak during test runs where we directly stimulated the neuron being recorded. Thus, we conclude that GlyT2 INs in the medial deep dorsal horn are presynaptic to Shox2 INs.

### Medial deep dorsal GlyT2GFP INs are heterogeneous in molecular markers expressed

Medial deep dorsal GlyT2GFP INs are comprised of relatively small subpopulations based on the expression of five molecular markers. Aside from the co-expressing Tfap2b and RORβ neurons, we found little overlap between RORβ expressing neurons and the other markers tested. Additionally, due to the observed expression pattern of Prox1 neurons being more medial than most Satb2, Tfap2b, and parvalbumin expressing neurons, there is unlikely to be significant co-expression of Prox1 and these markers. Other studies have shown little overlap of Satb2 with PV (Gradwell et al., 2024) and Tfap2b (Levine et al., 2014) in deep dorsal horn INs. Together this demonstrates that the molecular markers label mainly distinct populations of medial deep dorsal GlyT2GFP INs. With the markers tested, we can account for approximately half of the total medial deep dorsal GlyT2GFP IN population. Other markers such as Sema5b (Russ et al., 2021) and Pou6f2 (Russ et al., 2021; Bikoff et al., 2016) have been shown to label inhibitory neurons in the deep dorsal horn and may comprise part of the uncharacterized population of medial deep dorsal GlyT2GFP INs.

While the molecular markers tested label inhibitory neurons in the medial deep dorsal horn, none of the markers are exclusive to this region. All markers tested are expressed more broadly medial-laterally and label neurons in other laminae of the spinal cord. In addition to glycinergic neurons, GABAergic neurons have also been shown to express RORβ, Satb2, Tfap2b, and parvalbumin (Koch et al., 2017; Hilde et al., 2016; Levine et al., 2014; Gradwell et al., 2024), and a dorsal subpopulation of RORβ neurons is glutamatergic (Koch et al., 2017). Although it is of great interest to specifically manipulate medial deep dorsal GlyT2 INs, or subpopulations thereof, to determine their functional role, targeting a subpopulation with any of the molecular markers tested would not be sufficient for such experiments due to their lack of specificity to the medial deep dorsal GlyT2 population.

### Proposed functional role of medial deep dorsal GlyT2 INs

We demonstrated that medial deep dorsal GlyT2GFP INs receive monosynaptic input from sensory afferents. While these experiments do not conclusively show which afferent fiber types are providing this input, the robust parvalbumin fiber staining coincident with the medial deep dorsal GlyT2GFP INs suggests that it is at least in part from proprioceptive afferents (Alvarez et al., 2004; de Nooij et al., 2013). The positioning of this population of GlyT2 INs in the medial deep dorsal horn is optimal for multimodal sensory afferent integration (Jankowska, 2022; Stachowski and Dougherty, 2021; Abraira and Ginty, 2013). A population of parvalbumin^+^ GlyT2 INs in the medial deep dorsal horn has been shown to receive both proprioceptive and cutaneous input (Gradwell et al., 2024), supporting a possible role for integration of multiple afferent types for the GlyT2 population identified in this study. Additionally, most of the GlyT2GFP INs we recorded from received afferent input from multiple segments of the lumbar cord. By receiving afferent input from multiple lumbar dorsal roots, these neurons could act as a convergence point and integrate information, not only from multiple sensory afferent types, but also from multiple hindlimb muscles. Integration of this information could support adaptive locomotion and provide adjustments depending on environmental context (Markin et al., 2010). Additionally, since a population of Shox2 INs is involved in rhythm generation (Dougherty et al., 2013), the medial deep dorsal GlyT2 INs could be involved in the regulation of the phase transitions from stance and swing (Lam and Pearson, 2002). Due to their input and output connectivity, medial deep dorsal GlyT2 INs likely act as a convergence point for sensory afferent input to locomotor Shox2 INs, that may interrupt or reinforce the locomotor rhythm.

After chronic SCI, the inhibitory sensory afferent pathway to Shox2 INs is lost (Garcia-Ramirez et al., 2021) and with this study we have identified a population of GlyT2GFP INs that are likely involved in these SCI-induced changes. Given the present data, medial deep dorsal GlyT2GFP INs regulate low-threshold afferent input to locomotor Shox2 INs. Proprioceptive afferent feedback is crucial for both the initiation and maintenance of locomotor recovery after SCI, highlighting the importance for proper modulation of this input (Takeoka and Arber, 2019; Takeoka et al., 2014; Formento et al., 2018). Therefore, it is possible that the loss of the medial deep dorsal GlyT2 IN input to Shox2 INs in chronic SCI contributes to locomotor deficits, and restoring the inhibitory sensory afferent pathway to Shox2 INs could result in functional improvements.

The molecular marker profile of medial deep dorsal GlyT2GFP INs is informative to the possible function of this population. Tfap2b, RORβ, Satb2, and parvalbumin expressing neurons have various functional roles, including encoding motor synergies (Levine et al., 2014), presynaptic inhibition (Koch et al., 2017), nociceptive withdrawal reflexes (Hilde et al., 2016), and modulation of gait dynamics (Gradwell et al., 2024), respectively. While their role in manifesting sensorimotor behaviors differs, they share commonalities. Subpopulations of Tfap2b, RORβ, Satb2, and parvalbumin expressing neurons in the deep dorsal horn receive proprioceptive afferent input and target ventral premotor and/or motor networks (Hilde et al., 2016; Levine et al., 2014; Koch et al., 2017; Gradwell et al., 2024). This aligns with the input and output connectivity we demonstrated for the medial deep dorsal GlyT2GFP INs. Combining their synaptic connectivity with their molecular profile, it is likely that medial deep dorsal GlyT2 INs are involved in modulating proprioceptive afferent input to Shox2 INs to facilitate sensorimotor behaviors.

### Sensory innervation modality as a functional determinant over molecular signature in the dorsal horn

Due to the heterogeneity of the molecular identity of medial deep dorsal GlyT2GFP INs, it is possible that laminar position, and therefore the type of afferent input they receive, may be more indicative of their function. Sensory afferents conveying different modalities terminate in the dorsal horn with a laminar organization and this strongly influences the function of the neurons within each lamina (Gatto et al., 2019; Smith and Ross, 2020). Recently, it has been demonstrated in the superficial dorsal horn, that molecularly heterogenous excitatory INs drive various sensorimotor reflexes depending on their laminar location (Gatto et al., 2021; Peirs et al., 2021). This supports that the topographic organization of sensory afferent termination is one of the key determinants of function for INs in the dorsal horn, and in this case is more informative than the molecular signature of the INs (Gradwell and Abraira, 2021). GlyT2GFP INs presynaptic to Shox2 INs are spatially restricted to the medial deep dorsal horn but are comprised of subsets of neurons expressing multiple molecular markers. This suggests that, like excitatory INs, inhibitory INs may organize functionally based on afferent innervation patterns. In the case of medial deep dorsal GlyT2 INs, the robust overlap with proprioceptive afferent terminals suggests this input is a defining feature of their function. Combining this with their synaptic connections to locomotor Shox2 INs, this defines medial deep dorsal GlyT2 INs based on input and output connectivity, positioning them to regulate sensory afferent input to motor circuitry. The addition of the complexity of multiple molecular markers may then allow for a level of specificity or a redundancy within the system.

## Data Availability

The original contributions presented in this study are included in this article/Supplementary material, further inquiries can be directed to the corresponding author.
