# Supplementary material for: Molecularly diverse glycinergic interneurons in the medial deep dorsal horn are interposed between primary sensory afferents and spinal Shox2 interneurons
Source: Front Neural Circuits. 2026 Sep 3;20:1854977. doi: 10.3389/fncir.2026.1854977 (PMC13582541; doi:10.3389/fncir.2026.1854977)
Supplement: Supplementary file 1 [file Data_Sheet_1.docx]

Supplementary Material

# 1 Supplementary Figures

**Supplementary Figure 1.** Expression patterns of all molecular markers tested in the lumbar spinal cord. (**A-E**) Representative images of lumbar spinal slices showing Tfap2b (**A**), parvalbumin (PV) (**B**), Satb2 (**C**), Prox1 (**D**), or RORβ (**E**), expression in red. GlyT2GFP neurons are shown in green. The white box shows the area defined as the medial deep dorsal horn.

**Supplementary Figure 2.** A sub-population of medial deep dorsal GlyT2GFP INs express both RORβ and Tfap2b. A Tfap2b antibody was applied to lumbar spinal tissue from *RORβ::Cre;R26-lsl-tdTomato;GlyT2GFP* mice. Arrows point to GFP^+^tdTomato^+^Tfap2b^+^ neurons in the medial deep dorsal horn. Scale bars are 50µm.

**Supplementary Figure 3.** Excitatory currents evoked in Shox2 INs from kainate stimulation were observed when attempting to test inhibitory neurons in lamina III and VI-VII. **(A)** Top: Individual sweeps of voltage clamp recordings at -20mV from a Shox2 IN when a cluster of GAD67 INs is stimulated using kainate. The duration of the kainate stimulation is denoted by the orange bar. Bottom: The average of the trials recording spontaneous activity of the Shox2 IN in the absence of kainate is shown in gray, and the mean of evoked EPSCs is shown in the black trace. **(B)** A representative spinal slice showing the location of putative excitatory INs when targeting inhibitory neurons in GAD67GFP mice (blue) or GlyT2GFP mice (green) and the paired Shox2 IN (red).
